# Communication-Efficient Federated Class-Incremental Intrusion Detection for Edge IoT Networks

**DOI:** 10.3390/s26144630

**Published:** 2026-07-21

**Authors:** Ziang Wu, Buzhen He, Zhiwei Si, Chen Qiu, Xiuheng Liao, Chunhua Su

**Affiliations:** 1 Graduate School of Computer Science and Engineering, University of Aizu, Aizu-Wakamatsu 965-8580, Japan; d8262105@u-aizu.ac.jp (Z.W.); d8272107@u-aizu.ac.jp (Z.S.); chsu@u-aizu.ac.jp (C.S.); 2 School of Computer Science and Artificial Intelligence, Lanzhou University of Technology, Lanzhou 730050, China; 231080292006@lut.edu.cn; 3 Iwate Biotechnology Research Center, Kitakami 024-0003, Japan; c-qiu@ibrc.or.jp

**Keywords:** Internet of Things, federated learning, class-incremental learning, intrusion detection, communication compression, Edge IoT

## Abstract

The continuous emergence of new attack classes challenges intrusion detection in edge Internet of Things (IoT) networks. Although federated learning enables distributed devices to collaboratively train a shared detector without exchanging raw traffic data, most federated intrusion detection systems assume a fixed label space. Retraining with all historical data incurs substantial storage and computation costs, whereas updating only with newly collected samples can cause catastrophic forgetting. The detector must mitigate catastrophic forgetting of previously observed attack classes while preserving sufficient new-class plasticity to learn emerging attacks under highly non-IID device data, intermittent client availability, constrained local memory, and repeated communication over bandwidth-limited and intermittently connected links. To address these challenges, this paper proposes EdgeFedCIL, a communication-efficient federated class-incremental intrusion detection framework. EdgeFedCIL preserves historical knowledge through client-local replay and knowledge distillation while reducing repeated model transmission through adaptive low-rank compression, quantization, and error feedback. A classifier-head protection strategy further limits compression-induced degradation of class discrimination. Experiments on public intrusion-detection datasets show that EdgeFedCIL achieves competitive or superior detection and historical-knowledge retention performance, particularly under highly heterogeneous client distributions, while reducing cumulative client-to-server model transmission by up to approximately 10.54 times relative to full-precision transmission. These results demonstrate the effectiveness of EdgeFedCIL for continual and communication-efficient intrusion detection in resource-constrained edge IoT networks.

## 1. Introduction

The proliferation of Internet of Things (IoT) devices has expanded the attack surface of edge networks. Heterogeneous sensors, gateways, industrial devices, and local services are increasingly exposed to diverse cyber threats. Successful attacks may compromise sensitive information, disrupt network services, or manipulate connected physical processes. Intrusion detection systems (IDS) are therefore essential for identifying malicious traffic and protecting edge IoT environments [1,2]. Learning-based IDS are particularly valuable because they can capture complex traffic patterns that are difficult to represent using manually defined signatures, thereby improving the detection of diverse and previously unseen attacks [3].

Effective learning-based intrusion detection requires representative traffic collected from different devices, network segments, and administrative domains. Directly centralizing such traffic is often impractical because network traces may contain sensitive user, device, and operational information. Transmitting large volumes of raw traffic also introduces substantial bandwidth and storage overhead in resource-constrained edge IoT environments [4]. Federated learning (FL) enables distributed clients to collaboratively train a shared model while retaining their original traffic records locally [5,6]. Keeping raw records local reduces their direct exposure, but does not by itself provide formal protection against information leakage from model updates or malicious participants. FL-based intrusion detection has therefore become an important approach for data-local collaborative security across IoT, industrial, and vehicular edge networks [7,8,9,10].

Nevertheless, conventional FL-based IDS generally assume that the attack label space remains fixed throughout training. This assumption is unsuitable for long-running edge IoT systems, where new vulnerabilities, malware variants, device behaviors, and attack strategies may emerge after deployment. Retraining the global detector using all previously collected traffic whenever a new attack class appears requires clients to retain an ever-growing historical dataset and repeatedly perform costly optimization. Conversely, updating the detector using only newly collected samples can cause catastrophic forgetting, whereby learning new attacks substantially degrades the recognition of previously observed classes [11,12]. Federated class-incremental learning (FCIL) is therefore needed to enable distributed detectors to sequentially acquire emerging attack classes while continuing to recognize the classes learned in earlier phases.

Applying FCIL to edge IoT intrusion detection introduces several coupled challenges. First, the detector must balance stability for historical classes with sufficient plasticity for newly introduced classes [13]. This balance becomes more difficult when client data are non-IID, new attack classes are unevenly distributed across clients, local exemplar memory is limited, and only a subset of clients participates in each communication round [14,15]. Second, federated optimization is repeated in every incremental phase, causing communication costs to accumulate across both phases and rounds. This burden can make continual model updating impractical over bandwidth-limited or intermittently connected links [16]. Third, generic update compression may distort information required for retaining historical classes or distinguishing newly introduced attacks. These coupled requirements motivate a framework that coordinates continual-learning objectives with communication-efficient federated optimization.

To address these challenges, we propose EdgeFedCIL, a communication-efficient FCIL framework for edge IoT intrusion detection. At the learning level, EdgeFedCIL employs a unified continual-learning objective supported by a bounded client-local exemplar memory. Feature-space herding retains representative historical samples, while new-class-weighted classification promotes adaptation to emerging attacks and old-class knowledge distillation constrains deviations from previously acquired knowledge. At the communication level, client model differences are represented through layer-wise adaptive low-rank approximation and low-bit quantization. The retained rank is determined by the spectral-energy distribution of each update, allowing the transmitted representation to follow the information structure of individual layers. Client-specific error feedback reintroduces discarded update information in subsequent communication rounds, and classifier-head protection reverts the classification layer to full-precision transmission when its relative reconstruction error exceeds a predefined threshold. Through this learning-and-communication co-design, EdgeFedCIL balances historical-class retention, new-class plasticity, bounded local memory, and cumulative communication cost under non-IID data and partial client participation.

The main contributions of this work are summarized as follows:

- We propose EdgeFedCIL, a federated CIL framework designed to continually learn emerging attack classes in edge IoT environments.
- We develop a federated continual-learning algorithm with a unified client-local objective supported by adaptive exemplar replay. New-class-weighted learning promotes adaptation to emerging attacks, while old-class knowledge distillation constrains the degradation of previously acquired knowledge. This design mitigates catastrophic forgetting while preserving new-class plasticity, enabling distributed detectors to continuously adapt to evolving intrusion threats in edge IoT environments.
- To address the communication constraints of edge IoT systems, we design a communication mechanism based on spectral-energy-adaptive low-rank approximation. Low-bit quantization reduces the representation cost, client-specific error feedback carries discarded update information across communication rounds, and reconstruction-error-based classifier-head protection limits distortion of class-discriminative parameters. This coordinated mechanism substantially reduces cumulative client-to-server transmission while preserving the update information required for continual learning.
- Extensive experiments on public intrusion-detection datasets representative of edge IoT environments demonstrate that the proposed framework achieves competitive or superior detection and historical-knowledge retention performance, particularly under highly heterogeneous client distributions, while substantially reducing client-to-server communication.

The remainder of this paper is organized as follows. Section 2 reviews the related work. Section 3 presents the system model and problem formulation. Section 4 introduces the proposed framework, while Section 5 describes the model-update compression and aggregation procedure. Section 6 presents the experimental evaluation, Section 7 discusses scalability and limitations, and Section 8 concludes the paper.

## 2. Related Work

### 2.1. Intrusion Detection Systems

Traditional signature-based IDS match observed traffic against predefined attack patterns [17], whereas anomaly-based IDS identify deviations from learned normal behavior [18]. Signature-based methods are effective for known threats, but their reliance on manually maintained rules limits their ability to detect previously unseen attacks. Data-driven IDS have therefore increasingly adopted deep neural networks to model nonlinear and temporal traffic characteristics [19]. Such capabilities are particularly important in edge IoT networks, where heterogeneous devices generate diverse traffic patterns and may be exposed to rapidly evolving attacks. Lin et al. [20] proposed a time-related intrusion detection model that integrates stacked sparse autoencoders with recurrent neural networks. The autoencoders learn compact traffic representations, while the recurrent component captures temporal dependencies among network events. He et al. [21] developed a multimodal sequential approach that integrates multi-view traffic features through deep autoencoders and a long short-term memory (LSTM) network, enabling the detector to exploit complementary information from different feature groups. Hu et al. [22] proposed a deep one-class intrusion detection scheme for software-defined industrial networks. Their method extracts network-state features, reduces redundant dimensions, and learns anomaly scores using only normal operating data. Although these approaches improve traffic representation and attack recognition, they are mainly trained in a centralized manner. Traffic collected from edge devices, gateways, and industrial sites must therefore be transferred to a common location. Such data movement is often impractical in edge IoT environments because of privacy concerns, limited backhaul capacity, and the need for low-latency local responses. Moreover, these methods generally assume that the training distribution and attack label space remain stable after deployment, which is inconsistent with the continuously evolving operating conditions of edge IoT systems.

### 2.2. Federated Learning

FL enables multiple clients to collaboratively optimize a global model while retaining raw data at their local collection points [5,23,24]. This paradigm is well suited to distributed intrusion detection across edge gateways, vehicles, industrial sites, and administrative domains because it reduces the need to centralize sensitive traffic records. Beyond basic distributed training, existing FL research has extensively investigated aggregation strategies, personalization, optimization, robustness, network topology, data heterogeneity, privacy, and security threats [25,26].

Communication efficiency is another major research direction because clients and the server repeatedly exchange high-dimensional model parameters or updates. PowerSGD uses power iteration to construct low-rank representations of matrix-shaped updates, thereby reducing the amount of transmitted information [27]. Top-K sparsification retains only the update coordinates with the largest magnitudes and omits the remaining entries from transmission [28]. In addition to directly compressing model updates, knowledge distillation has been introduced into FL to transfer predictive knowledge between models with different capacities. For example, FedGKT trains lightweight models on resource-constrained edge devices and transfers their knowledge to a larger server-side model through knowledge distillation [29]. These studies establish communication compression and knowledge transfer as important foundations for resource-aware federated optimization.

Existing federated IDS studies have explored different local models, coordination architectures, and privacy mechanisms. Mothukuri et al. [7] developed a federated anomaly-detection framework for IoT security attacks. Their approach trains local detectors on distributed IoT data and aggregates the resulting updates to improve attack recognition without centralizing the original records. Liu et al. [8] combined FL with blockchain for collaborative intrusion detection in vehicular edge computing. They used blockchain to coordinate distributed training and introduced secure model-upload and model-quality evaluation mechanisms to improve aggregation trustworthiness. Ruzafa-Alcázar et al. [9] investigated privacy-preserving FL for industrial IoT intrusion detection. They evaluated Gaussian- and Laplace-based differential privacy mechanisms under different federated configurations to examine the trade-off between privacy protection and detection performance.

These methods reduce direct traffic sharing and improve collaborative detection across distributed edge environments. However, most of them are designed for one-time training with a fixed label space. They determine where data are stored and how local models are aggregated, but do not explicitly address how an edge IoT detector should learn emerging attack classes without forgetting previously learned threats. Existing communication-efficient and knowledge-distillation-based FL methods are likewise generally developed for stationary learning tasks rather than evolving attack-label spaces. Moreover, they do not fully consider the repeated communication burden introduced when federated training is executed across multiple incremental phases.

### 2.3. Class-Incremental Learning

Class-incremental learning (CIL) enables a model to learn newly introduced classes while retaining the ability to recognize classes learned in previous phases. Existing CIL methods mainly mitigate catastrophic forgetting through regularization, knowledge distillation, exemplar rehearsal, or architectural expansion. Learning without Forgetting (LwF) uses knowledge distillation to preserve the output responses of the previous model while learning new classes [30]. Rebuffi et al. [31] proposed incremental classifier and representation learning (iCaRL), which retains representative exemplars of previously learned classes and uses them to support knowledge preservation. Yan et al. [32] introduced Dynamically Expandable Representation (DER), which allocates additional representation components for new classes while preserving previously learned features.

Although these methods provide effective mechanisms for reducing catastrophic forgetting, they are primarily designed for centralized learning. Their direct application to federated environments is challenging because historical samples and newly introduced classes may be unevenly distributed across clients, while local models are periodically aggregated into a shared global model. These factors can cause forgetting at both the client and global levels.

Dong et al. [33] proposed Global-Local Forgetting Compensation (GLFC) for federated class-incremental learning. At the local level, GLFC introduces a class-aware gradient compensation loss to reduce the bias caused by imbalanced old- and new-class samples and employs class-semantic relation distillation to preserve inter-class relationships. At the global level, a proxy server selects a suitable previous global model to assist local knowledge distillation, thereby alleviating forgetting caused by non-IID class distributions across clients.

Luo et al. [34] proposed Federated Class-Incremental Learning with PrompTing (FCILPT), a rehearsal-free method that preserves task-related and task-independent knowledge using prompt pools. Before global aggregation, FCILPT aligns the task information represented by prompts across clients to reduce inconsistencies caused by missing classes and non-IID local data. Unlike exemplar-based FCIL methods, FCILPT relies on a frozen pretrained vision transformer and updates prompt parameters rather than the complete backbone model.

Federated incremental intrusion-detection studies have also investigated knowledge preservation and model aggregation under evolving and heterogeneous traffic distributions [35,36,37]. However, existing CIL and FCIL methods primarily focus on mitigating catastrophic forgetting. They generally do not consider the cumulative communication overhead caused by repeatedly transmitting model updates across multiple incremental phases. Moreover, generic communication compression is rarely designed together with the stability–plasticity requirements of federated class-incremental intrusion detection. EdgeFedCIL addresses this gap by coordinating continual knowledge preservation with adaptive client-to-server update compression under non-IID incremental traffic streams.

## 3. System Model and Problem Formulation

This section formalizes the edge IoT federated CIL-based IDS setting considered in this work. We first describe the federated system architecture and the class-incremental label space. We then define the non-repetitive phase stream, the non-IID client partition, partial client participation, and the common client-local balancing rule. Finally, we present the generic federated optimization process and formulate the learning objective and practical constraints.

The principal notation used throughout this paper is summarized in Table 1. Symbols used only within an individual equation are defined locally when they first appear.

### 3.1. Edge IoT Federated IDS

We consider an edge-assisted IDS consisting of one coordinating server, denoted by S, and *K* distributed edge clients indexed by k∈{1,2,…,K}. A client may represent an IoT gateway, an industrial edge node, or a local security appliance that monitors the traffic generated by a group of connected devices.

A processed traffic sample is represented by (x,y), where $x\in R^{d}$ is a *d*-dimensional traffic-feature vector and *y* is its traffic label. The label may correspond to benign traffic or an attack category. All clients use the same feature schema and model architecture, while their sample quantities and label proportions may differ substantially.

Raw traffic records remain on the clients throughout FL. The server initializes the global model, selects participating clients, broadcasts the current parameters, and aggregates the local updates returned by the selected clients. Client-local traffic records and retained exemplars are not directly uploaded to the server.

This study assumes an honest coordinating server and benign participating clients that follow the prescribed training and aggregation protocol. Under this threat model, data locality reduces the direct exposure of sensitive traffic records but does not provide a formal privacy or security guarantee for the exchanged model updates. Malicious-client attacks, model poisoning, backdoor injection, gradient inversion, and server compromise are outside the scope of this study. Robust aggregation, secure aggregation, differential privacy, and malicious-update detection may be incorporated as complementary defenses in future work.

The learning process contains *T* sequential class-incremental phases, each of which contains multiple synchronous federated communication rounds. At round *r* of phase *t*, the server broadcasts the current global parameters $\theta_{t,r}$ to the participating clients. Each selected client performs local optimization and returns a model update, after which the server produces the next global model through weighted aggregation.

### 3.2. Federated CIL Data Stream

#### 3.2.1. Class-Incremental Label Space and Evaluation Scope

In a dynamic IoT environment, previously unseen attacks may emerge after an intrusion detection model has already been deployed. Accordingly, training samples from future attack classes are unavailable before their first-arrival phases. For controlled class-incremental evaluation, however, the complete selected label space and the corresponding classifier output dimension are fixed in advance.

Let C denote the complete label space considered by the learning system. The classes introduced for the first time in phase *t* are denoted by $C_{t}^{\mathrm{new}}$. A class is assigned to exactly one first-arrival phase, so the new-class sets of different phases do not overlap. By the end of phase *t*, the observed label space is denoted by $C_{t}^{\mathrm{seen}}={⋃}_{\tau =1}^{t}C_{\tau }^{\mathrm{new}}$. For t>1, classes observed before the current phase are treated as historical classes and are denoted by $C_{t}^{\mathrm{old}}={⋃}_{\tau =1}^{t-1}C_{\tau }^{\mathrm{new}}$, whereas $C_{1}^{\mathrm{old}}=⌀$ in phase 1.

The system follows a single-head, task-agnostic class-incremental protocol. A fixed classifier head covers the complete selected label space C. During phase *t*, the training stream and the evaluation data contain only classes in $C_{t}^{\mathrm{seen}}$; however, output dimensions associated with not-yet-introduced classes are not explicitly masked during loss computation or prediction. During inference, neither the phase identity nor the first-arrival phase of a test sample is provided to the model.

After phase *t*, the selected global model is evaluated over all classes observed so far. It must acquire the newly introduced classes while preserving its detection capability for historical classes.

#### 3.2.2. Non-Repetitive Decay-Based Stream Construction

Realistic edge IoT systems continuously generate distributed traffic data, while device behaviors, service conditions, and traffic compositions evolve over time [38,39]. When a new attack class emerges, traffic from previously observed classes does not disappear immediately. Instead, newly emerging and historical classes may coexist in the incoming stream, while their relative prevalence changes over time. A strict class-incremental protocol that completely removes an old class after its first-arrival phase therefore oversimplifies this operational pattern. Conversely, repeatedly presenting the complete historical training set introduces unrealistic sample reuse and makes the setting resemble ordinary joint retraining rather than continual learning.

To approximate this evolving mixture of new and historical traffic in a controlled and reproducible manner, we construct a non-repetitive decay-based stream. A class contributes its largest allocation when it first appears and progressively smaller allocations of its remaining unused real samples in subsequent phases. The decay rule models the gradual reduction in the prevalence of previously introduced classes; it is used as a controlled approximation rather than an assumption that all real edge IoT traffic follows an exact exponential arrival distribution.

Let $D_{c}^{\mathrm{tr}}$ denote the complete real training pool of class *c*, and let $s_{c}$ be the phase in which class *c* is introduced for the first time. For phase $t\geq s_{c}$, its release weight is defined as $w_{c,t}=\gamma^{t-s_{c}}$, where 0<γ<1. The normalized allocation coefficient is

(1) $$\xi_{c,t}=\frac{\gamma^{t-s_{c}}}{\sum_{\tau =s_{c}}^{T}\gamma^{\tau -s_{c}}}.$$

The complete benchmark horizon *T* is specified before stream construction so that the normalized allocation coefficients can be determined. This information is used only to construct the controlled benchmark stream; during training, samples assigned to future phases remain unavailable until their corresponding phases begin. Let $N_{c}=\left|D_{c}^{\mathrm{tr}}\right|$ denote the number of real training samples of class *c*. The real-valued quota for phase *t* is $N_{c}\xi_{c,t}$. Each quota is first rounded down, after which the remaining samples are assigned one at a time to the phases with the largest fractional remainders. Ties are resolved according to phase order. The complete class-specific pool is shuffled once using the fixed experimental seed and then sequentially divided according to the resulting integer quotas. Consequently, every real sample is assigned to exactly one phase, and no sample is duplicated or omitted across the complete stream.

The stream of phase *t* is formed by combining the samples allocated to that phase from all classes that have appeared by phase *t*. Newly introduced classes generally contribute the largest allocations, whereas previously introduced classes contribute smaller sets of previously unused samples. Thus, the stream represents the coexistence of emerging and historical traffic without repeatedly replaying the same real records. The term new class refers to a class in its first-arrival phase, whereas an old-class stream sample is a previously unused real sample belonging to a class introduced in an earlier phase. The identical phase stream is used by EdgeFedCIL and all comparison methods.

### 3.3. Non-IID Client Partition and Partial Participation

The phase stream is distributed across clients using a class-wise Dirichlet partition with concentration parameter α. For each class represented in phase *t*, a client proportion vector is sampled from a symmetric Dirichlet distribution over the *K* clients. The parameter α>0 controls the degree of label skew: smaller values concentrate samples of a class on fewer clients and therefore produce stronger statistical heterogeneity, whereas larger values lead to more balanced class proportions across clients.

The sampled proportions determine how the real samples of each class are divided among the *K* clients. Each phase sample is assigned to exactly one client, and the union of all client-local partitions reconstructs the complete phase stream without duplication or omission. Let $D_{k,t}$ denote the resulting real stream samples held by client *k* in phase *t*.

Client distributions can differ both spatially and temporally. Spatial heterogeneity means that clients within the same phase may have different class proportions, sample quantities, and feature distributions. Temporal heterogeneity means that the distribution of an individual client may change across consecutive phases as new attack classes appear and local traffic composition evolves.

The system also adopts partial client participation. Given a participation ratio ρ∈(0,1], the server samples m=max{1,round(ρK)} clients without replacement in each communication round. The selected subset may change from round to round, while the total client population remains fixed. A selected client with no usable local optimization samples does not contribute an update in that round. Partial participation represents intermittent connectivity and limited resource availability at edge devices, thereby avoiding the requirement that the complete client population remain active simultaneously.

To ensure controlled comparisons, the phase stream, Dirichlet allocation, and round-wise client-selection schedule are fixed for each experimental setting and reused across all methods.

### 3.4. Common Client-Local Class Balancing

The combination of class-incremental release and Dirichlet partitioning can leave a participating client with severe local class imbalance. To reduce optimization collapse toward locally dominant classes, every compared method applies the same constrained balancing rule after constructing its own pre-balancing local optimization set. For EdgeFedCIL, this set contains current real stream samples and retained real exemplars; for methods without replay, it contains only the current real stream samples.

Let $A_{k,t}$ denote the distinct real samples in the client-local optimization set before balancing, and let $n_{k,t}^{c}$ be the number of samples from class *c* in this set. The local majority count is the largest positive class count, denoted by $n_{k,t}^{\max}$. For a target ratio $\eta_{\mathrm{bal}}\in \left(0,1\right]$ and a maximum expansion factor κ≥1, the target count of class *c* is

(2) $${\hat{n}}_{k,t}^{c}=\max\;\left\{n_{k,t}^{c},\min\;\left[\left\lfloor \eta_{\mathrm{bal}}n_{k,t}^{\max}\right\rfloor ,\left\lfloor \kappa n_{k,t}^{c}\right\rfloor \right]\right\}.$$

This rule raises a minority class toward a fraction $\eta_{\mathrm{bal}}$ of the local majority size while preventing its sample count from exceeding κ times the original count. The outer maximum ensures that the operation never removes existing samples. This two-sided restriction avoids both ineffective balancing and excessive synthetic expansion.

When a class requires expansion and contains at least two local samples, Synthetic Minority Over-sampling Technique (SMOTE) generates additional samples through within-class interpolation:

(3) $$\tilde{x}=x_{i}+\lambda \left(x_{j}-x_{i}\right),\;\lambda \in \left[0,1\right],$$

where $x_{j}$ is selected from the nearest same-class neighbors of $x_{i}$. If a target minority class contains only one local sample, random oversampling is used because no interpolation pair can be formed. Balancing is skipped for a single-class client or when no class requires expansion.

Let $B_{k,t}$ denote the client-local optimization multiset after balancing. It contains the distinct real samples in $A_{k,t}$ together with any synthetic or duplicated samples generated to approach the target counts in (2). If balancing is skipped, then $B_{k,t}=A_{k,t}$.

Synthetic samples exist only inside the current client-local optimization process. They are not added to the non-repetitive real stream, retained as exemplars, transmitted to the server, or counted when determining sample-weighted aggregation coefficients. Therefore, balancing changes the local mini-batch distribution without changing the ownership, release phase, or communication accounting of real traffic records. The concrete values of $\eta_{\mathrm{bal}}$ and κ are reported in Section 6.1.

### 3.5. Federated Optimization Under Heterogeneous Data

Let $f_{\theta }:R^{d}\to R^{\left|C\right|}$ denote the intrusion detection model parameterized by θ. For a client with a nonempty local dataset, a generic empirical objective in phase *t* is

(4) $$F_{k,t}\left(\theta \right)=\frac{1}{|D_{k,t}|}\sum_{\left(x_{i},y_{i}\right)\in D_{k,t}}\ell \;\left(f_{\theta }\left(x_{i}\right),y_{i}\right),$$

where ℓ(·,·) denotes the classification loss. This objective provides a common abstraction; a specific method may augment the local optimization set with retained exemplars, apply the balancing operator described above, or add regularization and knowledge-preservation terms.

At communication round *r*, every participating client initializes its local parameters with the current global model, that is, $\theta_{t,r}^{k,0}=\theta_{t,r}$. After local optimization, client *k* obtains $\theta_{t,r}^{k}$ and forms the model difference $\Delta \theta_{k,t,r}=\theta_{t,r}^{k}-\theta_{t,r}$.

The server updates the global model through weighted aggregation:

(5) $$\theta_{t,r+1}=\theta_{t,r}+\sum_{k\in S_{t,r}^{+}}a_{k,t,r}\Delta \theta_{k,t,r},$$

where $S_{t,r}^{+}$ contains the selected clients that return valid updates. The non-negative aggregation coefficients sum to one and are computed from the number of distinct real samples in each client’s pre-balancing optimization set. Consequently, synthetic samples do not increase a client’s influence on the global model.

After each communication round in phase *t*, the current global model is evaluated on the fixed validation subset containing all classes in $C_{t}^{\mathrm{seen}}$. The checkpoint with the highest seen-class validation accuracy is selected as the phase-level model $\theta_{t}^{*}$. If multiple checkpoints achieve the same validation accuracy, the checkpoint with the lowest validation cross-entropy loss is selected. Validation data are used exclusively for checkpoint selection and are not used for model optimization or final performance reporting. After completing all communication rounds, the selected checkpoint is restored and evaluated on the fixed held-out test set over all classes observed by phase *t*. All reported detection and forgetting metrics are computed from the held-out test set. The selected model also initializes the next class-incremental phase and, when applicable, serves as the frozen teacher model.

### 3.6. Problem Definition and Design Requirements

Federated CIL-based intrusion detection requires the global model to maintain two competing properties. It must retain sufficient plasticity to acquire newly introduced classes while preserving sufficient stability to maintain decision boundaries for historical classes.

Let $L_{t}^{\mathrm{seen}}\left(\theta_{t}^{*}\right)$ denote the detection loss of the selected global model over all classes observed by phase *t*, and let $G_{t}\left(\theta_{t}^{*}\right)$ denote the degradation in historical-class performance after completing phase *t*. The system-design objective is to learn a sequence of global models that jointly minimizes cumulative seen-class loss and historical-class degradation:

(6) $$\underset{{\left\{\theta_{t}^{*}\right\}}_{t=1}^{T}}{\mathrm{minimize}}\;\left(\sum_{t=1}^{T}L_{t}^{\mathrm{seen}}\left(\theta_{t}^{*}\right),\;\sum_{t=2}^{T}G_{t}\left(\theta_{t}^{*}\right)\right).$$

This is a multi-objective system goal rather than a single differentiable client loss. The first component measures overall detection capability over the classes observed so far, whereas the second captures catastrophic forgetting after new classes are learned.

The learning process is subject to several practical constraints. Raw client data and retained exemplars remain local. Only a subset of clients is available in each communication round. Client distributions are non-IID across both clients and phases. Local memory, computation, and communication must remain compatible with resource-constrained edge devices. In addition, common data handling operations, including stream construction, client partitioning, participation scheduling, and local class balancing, must be held fixed across compared methods.

Accordingly, the desired method should adapt to emerging attacks, preserve historical detection knowledge, remain robust under spatial and temporal heterogeneity, and reduce communication demand without directly sharing raw traffic records. EdgeFedCIL addresses these requirements through adaptive client-local exemplar preservation, stability–plasticity-aware local learning, and communication-efficient federated updates, as described in the next section.

## 4. Proposed Framework

To address catastrophic forgetting under the sequential arrival of attack classes, EdgeFedCIL equips the federated IDS with a client-local continual-learning mechanism. This section describes how historical knowledge is retained and reused during incremental training. The communication-compression mechanism applied to the resulting client updates is presented separately in Section 5.

### 4.1. Overall Framework

**Figure 1:**
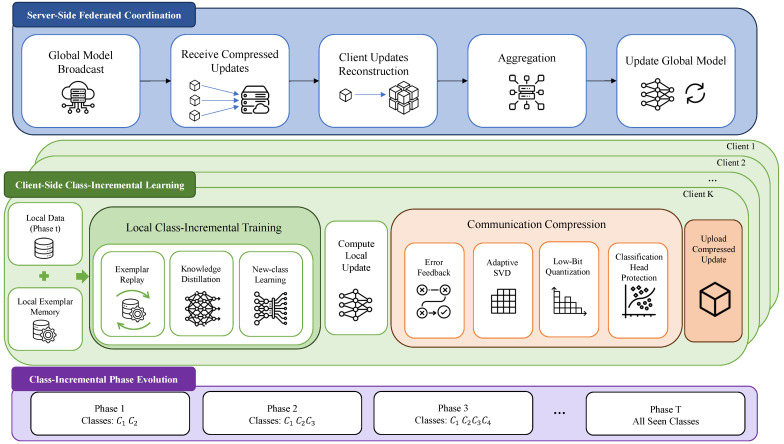
Overall framework of EdgeFedCIL.

Figure 1 illustrates the complete EdgeFedCIL workflow. In the first incremental phase, no historical exemplar or teacher model is available. Each selected client trains the received global model using its locally available real traffic samples. The resulting local update is passed to the communication module described in Section 5, and the server reconstructs and aggregates the transmitted updates. After the phase-level model has been selected using the common validation protocol, each client constructs its initial exemplar memory from the real samples observed locally in that phase.

From phase t>1, the selected model from phase t−1 initializes the current global model and is frozen as a teacher. Each participating client combines its current real traffic samples with the exemplars retained from previous phases, applies the common local balancing rule, and optimizes a student model under a joint classification and KD objective. At the end of the phase, the selected global model is used both to initialize the next phase and to update each client’s private exemplar memory.

The intrusion detection backbone is shared by EdgeFedCIL and all compared methods. The continual-learning contribution lies in the adaptive exemplar memory and the stability–plasticity-aware local objective described below, while the communication contribution is isolated in Section 5.

### 4.2. Client-Local Adaptive Exemplar Preservation

Retaining the complete historical traffic stream would cause local storage requirements to grow continuously. EdgeFedCIL instead maintains a bounded exemplar memory $M_{k,t}$ on client *k* after phase *t*. The memory contains only real traffic samples previously observed by that client and is never transmitted to the coordinating server.

#### 4.2.1. Stream-Safe Candidate Construction

At the end of phase *t*, client *k* prepares a class-specific candidate pool from two sources: the exemplars of that class retained after the previous phase and the real samples of the same class newly received in the current phase. Samples discarded in earlier phases cannot re-enter the pool because they are no longer locally available to the memory-update procedure. When a client receives no real sample in the current phase, its previous memory is carried forward unchanged. This construction preserves the sequential availability of the non-repetitive traffic stream and prevents the memory mechanism from silently accessing historical samples that should have disappeared.

#### 4.2.2. Adaptive Memory Budget and Class Allocation

Let $N_{k,t}^{\mathrm{obs}}$ denote the cumulative number of distinct real traffic samples observed by client *k* up to phase *t*, without recounting replayed exemplars. Let $C_{k,t}^{\mathrm{loc}}$ denote the number of classes represented in the client’s current candidate pools. The total exemplar budget is

(7) $$M_{k,t}=\min\left\{N_{k,t}^{\mathrm{obs}},M_{\max},\max\left[\left\lfloor \beta N_{k,t}^{\mathrm{obs}}\right\rfloor ,m_{\min}C_{k,t}^{\mathrm{loc}}\right]\right\},$$

where β is the memory ratio, $m_{\min}$ is the desired minimum number of exemplars per locally represented class, and $M_{\max}$ is the client-level storage cap. The budget therefore increases with the amount of locally observed real traffic, while never exceeding either the available samples or the device capacity.

The available budget is distributed approximately evenly among the locally represented classes. Each class quota is capped by the number of candidates actually available for that class. Allocation proceeds iteratively, and positions left unused by a class with an exhausted candidate pool are reassigned to other classes that still have unselected candidates. This procedure avoids reserving unusable memory slots and reduces domination by locally frequent classes.

#### 4.2.3. Feature-Space Herding

When the number of candidates for a class does not exceed its allocated quota, all candidates are retained. Otherwise, EdgeFedCIL applies feature-space herding. The selected global model of phase *t* extracts the representation immediately before the classifier head. For a sample x, the normalized representation is ${\bar{\phi }}_{t}\left(x\right)=\phi_{\theta_{t}^{*}}\left(x\right)/{∥\phi_{\theta_{t}^{*}}\left(x\right)∥}_{2}$. The mean of these normalized representations over the class-specific candidate pool is denoted by $\mu_{k,t}^{c}$.

At selection step *j*, let $R_{k,t,j}^{c}$ denote the candidates that have not yet been selected, and let the previously selected exemplars be $x_{1}^{*},\ldots ,x_{j-1}^{*}$. The next exemplar is chosen as

(8) $$x_{j}^{*}=\underset{x\in R_{k,t,j}^{c}}{\arg\min}{∥\mu_{k,t}^{c}-\frac{1}{j}\left({\bar{\phi }}_{t}\left(x\right)+\sum_{i=1}^{j-1}{\bar{\phi }}_{t}\left(x_{i}^{*}\right)\right)∥}_{2}.$$

Selection continues until the class quota is filled. The updated client memory consists of the exemplars selected for all locally represented classes. Because every client extracts features with the same selected global model, the memories are constructed in a common representation space, although the retained samples remain private and client-specific.

### 4.3. Stability–Plasticity-Aware Local Learning

At each incremental phase, the local learner must remain sufficiently plastic to acquire newly emerging attacks while remaining sufficiently stable to preserve historical decision boundaries. EdgeFedCIL addresses this trade-off through client-local exemplar replay, new-class-weighted classification, and old-class KD.

#### 4.3.1. Client-Local Exemplar Replay

Before local optimization, client *k* merges the current phase’s real traffic samples with the exemplars retained after phase t−1. Repeated real-sample indices are removed. The resulting number of distinct real samples is denoted by $n_{k,t}^{\mathrm{real}}$ and is recorded before synthetic balancing. The common client-local balancing rule in Section 3.4 is then applied, and mini-batches are drawn from the balanced optimization data. Synthetic samples influence local gradient computation only; they do not change $n_{k,t}^{\mathrm{real}}$, enter the exemplar memory, or affect the client’s aggregation weight.

#### 4.3.2. New-Class-Weighted Classification

For a mini-batch sample $\left(x_{i},y_{i}\right)$, let $o_{i}^{S}=f_{\theta }\left(x_{i}\right)$ denote the student logits. Consistent with the fixed-head protocol, the cross-entropy (CE) loss is computed over the complete output space C:

(9) $$\ell_{\mathrm{ce},i}=-\log\frac{\exp\left(o_{i,y_{i}}^{S}\right)}{\sum_{c\in C}\exp\left(o_{i,c}^{S}\right)}.$$

To improve adaptation to newly introduced attacks, EdgeFedCIL assigns weight $\omega_{\mathrm{new}}>1$ to samples whose labels belong to $C_{t}^{\mathrm{new}}$ when t>1, and weight 1 to all other samples. For a mini-batch $Z\subseteq B_{k,t}$, the weighted cross-entropy (WCE) loss is

(10) $$L_{\mathrm{WCE}}=\frac{1}{\left|Z\right|}\sum_{(x_{i},y_{i})\in Z}\omega_{t}\left(y_{i}\right)\ell_{\mathrm{ce},i},$$

where $\omega_{t}\left(y_{i}\right)=\omega_{\mathrm{new}}$ for a current new-class sample in phases t>1, and $\omega_{t}\left(y_{i}\right)=1$ otherwise. In phase 1, the loss reduces to ordinary CE because no historical class exists. Normalization by the mini-batch size |Z| allows the weighted loss to reflect both the relative importance and the local proportion of new-class samples. Consequently, mini-batches containing more newly introduced samples produce a stronger classification signal, which facilitates adaptation to emerging attacks. Historical-class retention is maintained through exemplar replay and old-class KD.

#### 4.3.3. Old-Class KD

At the beginning of phase t>1, the selected global model from phase t−1 is copied and frozen as the teacher, while the current trainable model acts as the student. For a mini-batch sample $x_{i}$, let $o_{i}^{T}=f_{\theta_{t-1}^{*}}\left(x_{i}\right)$ and $o_{i}^{S}=f_{\theta }\left(x_{i}\right)$ denote the teacher and student logits. KD considers only output dimensions associated with classes introduced before the current phase. For an old class *c*, the softened teacher and student probabilities are

(11) $$p_{i,c}^{T}=\frac{\exp(o_{i,c}^{T}/T_{\mathrm{kd}})}{\sum_{j\in C_{t}^{\mathrm{old}}}\exp\left(o_{i,j}^{T}/T_{\mathrm{kd}}\right)},\;p_{i,c}^{S}=\frac{\exp(o_{i,c}^{S}/T_{\mathrm{kd}})}{\sum_{j\in C_{t}^{\mathrm{old}}}\exp\left(o_{i,j}^{S}/T_{\mathrm{kd}}\right)},$$

where $T_{\mathrm{kd}}$ is the KD temperature. Let $p_{i,\mathrm{old}}^{T}$ and $p_{i,\mathrm{old}}^{S}$ collect these probabilities over $C_{t}^{\mathrm{old}}$. The batch-averaged KD loss, based on the Kullback–Leibler (KL) divergence, is

(12) $$L_{\mathrm{KD}}=\frac{T_{\mathrm{kd}}^{2}}{\left|Z\right|}\sum_{(x_{i},y_{i})\in Z}D_{\mathrm{KL}}\;\left(p_{i,\mathrm{old}}^{T}\;\;\|\;\;p_{i,\mathrm{old}}^{S}\right),$$

where $D_{\mathrm{KL}}\left(\cdot \;\|\;\cdot \right)$ denotes the KL divergence.

Restricting KD to historical outputs preserves relationships among previously learned attack categories without directly suppressing the output dimensions assigned to newly introduced classes. No teacher or KD term is used in phase 1.

#### 4.3.4. Joint Local Objective

The complete local objective is

(13) $$L_{k,t}=L_{\mathrm{WCE}}+\lambda_{\mathrm{kd}}L_{\mathrm{KD}}+\frac{\lambda_{2}}{2}{∥\theta ∥}_{2}^{2},$$

where $\lambda_{\mathrm{kd}}$ controls the contribution of KD and $\lambda_{2}$ is the parameter-regularization coefficient. Exemplar replay provides direct supervision for historical classes, new-class weighting promotes plasticity, and KD stabilizes the historical output structure.

### 4.4. Historical-Knowledge Stability Analysis

Exemplar replay and old-class KD provide complementary controls on historical-knowledge degradation. Consider a nonempty set $H_{k,t}\subseteq Z$ of historical exemplars in a local mini-batch. Define the average historical CE loss and old-output KL divergence as

(14) $${\bar{\ell }}_{k,t}^{\mathrm{old}}=\frac{1}{|H_{k,t}|}\sum_{\left(x_{i},y_{i}\right)\in H_{k,t}}\ell_{\mathrm{ce},i},\;{\bar{D}}_{k,t}^{\mathrm{old}}=\frac{1}{|H_{k,t}|}\sum_{\left(x_{i},y_{i}\right)\in H_{k,t}}D_{\mathrm{KL}}\;\left(p_{i,\mathrm{old}}^{T}\|p_{i,\mathrm{old}}^{S}\right).$$

**Proposition 1**  (Historical-sample stability). *For the historical exemplar set $H_{k,t}$, the empirical classification-error rate and the average teacher–student output drift satisfy*

(15) $$\begin{array}{cc} \frac{1}{|H_{k,t}|}\sum_{\left(x_{i},y_{i}\right)\in H_{k,t}}1\;\left\{\underset{c\in C}{\arg\max}\;o_{i,c}^{S}\neq y_{i}\right\}\; & \leq \frac{{\bar{\ell }}_{k,t}^{\mathrm{old}}}{\log2}, \end{array}$$

(16) $$\begin{array}{cc} \frac{1}{|H_{k,t}|}\sum_{\left(x_{i},y_{i}\right)\in H_{k,t}}{∥p_{i,\mathrm{old}}^{T}-p_{i,\mathrm{old}}^{S}∥}_{1}\; & \leq \sqrt{2{\bar{D}}_{k,t}^{\mathrm{old}}}. \end{array}$$

**Proof.**  For a misclassified historical sample, the student probability assigned to the true class is at most 1/2, and therefore $\ell_{\mathrm{ce},i}\geq \log2$. Averaging the resulting indicator bound gives (15). Pinsker’s inequality gives $∥p_{i,\mathrm{old}}^{T}-p_{i,\mathrm{old}}^{S}{∥}_{1}\leq \sqrt{2D_{\mathrm{KL}}\left(p_{i,\mathrm{old}}^{T}\|p_{i,\mathrm{old}}^{S}\right)}$. Averaging and applying Jensen’s inequality yields (16).    □

The first bound relates replay-supervised CE to empirical historical-class error, while the second shows how old-class KD limits teacher–student output drift. These guarantees apply to retained historical exemplars; forgetting on held-out traffic also depends on how well the bounded memory represents the historical data distribution.

### 4.5. Incremental Training Procedure

Algorithm 1 summarizes the CIL component of EdgeFedCIL. Communication encoding, reconstruction, and aggregation are delegated to Algorithm 2 in Section 5. The common validation protocol selects one checkpoint only after all scheduled rounds of a phase have been completed; it is not used for early stopping.

During phase 1, replay and KD are inactive because no historical information is available. From phase 2 onward, the previously selected global model serves as the frozen teacher, and each participating client jointly learns from current real traffic and private historical exemplars. Section 5 describes how each resulting model difference is compressed without changing this incremental-learning procedure.
**Algorithm 1** Federated CIL in EdgeFedCIL**Require:** Phase-wise client data, phase count *T*, communication rounds $R_{t}$, local epochs *E*
**Ensure:** Selected phase models $\theta_{1}^{*},\ldots ,\theta_{T}^{*}$
1:Initialize the global model and client memories2:**for** t=1 to *T* **do**3:     Initialize the phase model from $\theta_{t-1}^{*}$ if t>14:     Use $\theta_{t-1}^{*}$ as the frozen teacher if t>1; otherwise disable KD5:     Reset the phase-specific compression residuals6:     **for** r=1 to $R_{t}$ **do**7:         Select participating clients $S_{t,r}$8:         **for all** $k\in S_{t,r}$ in parallel **do**9:               $A_{k,t}\leftarrow \mathrm{unique}\left(D_{k,t}\cup M_{k,t-1}\right)$10:               Record $n_{k,t}^{\mathrm{real}}=\left|A_{k,t}\right|$ and construct $B_{k,t}$ using the local balancing rule11:               Train $\theta_{t,r}^{k}$ for *E* epochs using (13)12:        **end for**13:        Update $\theta_{t,r+1}$ using Algorithm 214:        Evaluate and record the current validation checkpoint15:    **end for**16:    Select the phase model $\theta_{t}^{*}$ using the common validation protocol17:    **for all** clients in parallel **do**18:          Update $M_{k,t}$ using (7) and (8); retain $M_{k,t-1}$ if no new real samples were observed19:     **end for**20:**end for**21:**return** $\theta_{1}^{*},\ldots ,\theta_{T}^{*}$


## 5. Communication-Efficient Update Compression

The communication module represents each client update using four complementary mechanisms. Adaptive low-rank approximation determines the amount of information retained for each matrix-shaped parameter according to its singular-value distribution, thereby avoiding a single fixed rank for all layers and rounds. Low-bit quantization further reduces the representation cost of the retained low-rank factors and one-dimensional parameters. Error feedback preserves the difference between the compensated update and its reconstructed representation, allowing information discarded in the current round to influence subsequent updates. Finally, classifier-head protection limits excessive distortion in the parameters that directly determine the decision boundaries among historical and newly introduced classes. Together, these mechanisms control update size while reducing the loss and accumulation of compression information during repeated federated communication.

### 5.1. Model Difference and Error Feedback

After local optimization in phase *t* and round *r*, client *k* forms the model difference $\Delta \theta_{k,t,r}=\theta_{t,r}^{k}-\theta_{t,r}$. For each parameter tensor *p*, the client maintains an independent residual from the previous communication round. Before compression, the tensor update is compensated as $u_{k,t,r}^{p}=\Delta \theta_{k,t,r}^{p}+e_{k,t,r-1}^{p}$.

Let ${\hat{u}}_{k,t,r}^{p}$ denote the update reconstructed locally from the representation that will be transmitted. The new residual is

(17) $$e_{k,t,r}^{p}=u_{k,t,r}^{p}-{\hat{u}}_{k,t,r}^{p}.$$

Residual buffers are reset at the beginning of each incremental phase and retained across its communication rounds. Consequently, information removed by compression in one round can be carried into a later update instead of being permanently discarded.

### 5.2. Spectral-Energy-Adaptive Low-Rank Approximation

For a nonzero two-dimensional compensated update $U^{p}\in R^{m_{p}\times n_{p}}$, EdgeFedCIL computes the singular value decomposition $U^{p}=A\Sigma V^{T}$. An all-zero update is represented directly as zero without performing the decomposition. Let the singular values of a nonzero update be ordered from largest to smallest. The retained rank is the smallest value whose cumulative squared singular values reach the prescribed spectral-energy threshold:

(18) $$\ell^{*}=\min\left\{\ell :\frac{\sum_{i=1}^{\ell }\sigma_{i}^{2}}{\sum_{i}\sigma_{i}^{2}}\geq \tau \right\},$$

where τ∈(0,1]. The resulting approximation is $U_{\ell^{*}}=A_{\ell^{*}}\mathrm{diag}\left(\sigma_{\ell^{*}}\right)V_{\ell^{*}}^{T}$. Because the selected rank depends on the spectrum of the current tensor, EdgeFedCIL adapts to differences across layers, clients, and communication rounds rather than enforcing one fixed rank globally.

The truncated singular value decomposition provides the minimum Frobenius-norm reconstruction error among all approximations with rank at most $\ell^{*}$. According to the rank-selection criterion in (18), the discarded spectral energy satisfies

(19) $${∥U^{p}-U_{\ell^{*}}^{p}∥}_{F}^{2}=\sum_{i>\ell^{*}}\sigma_{i}^{2}\leq \left(1-\tau \right){∥U^{p}∥}_{F}^{2}.$$

Therefore, τ controls the trade-off between the reconstruction accuracy and the transmitted rank. Increasing τ reduces the truncation error but generally increases the communication payload.

### 5.3. Low-Bit Quantization and Payload Accounting

For a real-valued tensor v, let $q_{\max}=2^{b-1}-1$. The scale used for symmetric *b*-bit quantization is defined as

(20) $$s\left(v\right)=\left\{\begin{array}{cc} \frac{\max|v|}{q_{\max}},\; & \max|v|>0,\\ 1,\; & \max|v|=0. \end{array}\right.$$

Each element is then mapped to an integer by

(21) $$Q_{b}\left(v\right)=\mathrm{clip}\left(\mathrm{round}\left(\frac{v}{s(v)}\right),-2^{b-1}+1,2^{b-1}-1\right),$$

and is reconstructed as $\hat{v}=s\left(v\right)Q_{b}\left(v\right)$. For a low-rank matrix update, the retained singular values use one tensor-level scale, the columns of $A_{\ell^{*}}$ use separate per-column scales, and the rows of $V_{\ell^{*}}^{T}$ use separate per-row scales. One-dimensional parameters are directly quantized with one tensor-level scale.

For a rank-$\ell^{*}$ matrix update of shape $m_{p}\times n_{p}$, the counted payload is

(22) $$B_{p}^{\mathrm{mat}}=b_{\mathrm{uv}}\left(m_{p}\ell^{*}+\ell^{*}n_{p}\right)+b_{s}\ell^{*}+32\left(2\ell^{*}+1\right),$$

where $b_{\mathrm{uv}}$ and $b_{s}$ are the bit widths used for singular vectors and singular values. The final term accounts for the floating-point scales. A directly quantized vector parameter *p* with $h_{p}$ elements requires $B_{p}^{\mathrm{vec}}=b_{v}h_{p}+32$ bits. These expressions count the transmitted representation rather than the size of a locally reconstructed dense tensor.

For comparison, transmitting the same $m_{p}\times n_{p}$ matrix update in full-precision floating-point format requires $32m_{p}n_{p}$ bits. The relative payload of the compressed representation is therefore

(23) $$\rho_{p}^{\mathrm{mat}}=\frac{B_{p}^{\mathrm{mat}}}{32m_{p}n_{p}}.$$

When $\ell^{*}\ll \min\left(m_{p},n_{p}\right)$, the dominant payload complexity decreases from $O(m_{p}n_{p})$ for dense transmission to $O(\ell^{*}\left(m_{p}+n_{p}\right))$ for the low-rank representation. The cumulative client-to-server upload over all incremental phases is

(24) $$B^{\mathrm{total}}=\sum_{t=1}^{T}\sum_{r=1}^{R_{t}}\sum_{k\in S_{t,r}^{+}}\sum_{p}B_{k,t,r}^{p},$$

where $B_{k,t,r}^{p}$ is determined by the selected representation of parameter tensor *p*. Consequently, cumulative upload grows linearly with the number of completed client transmissions and communication rounds, while the payload of each matrix-shaped parameter is controlled by its adaptively selected rank.

For a matrix-shaped update of size $m_{p}\times n_{p}$, the exact singular value decomposition requires $O\;\left(m_{p}n_{p}\min\left(m_{p},n_{p}\right)\right)$ operations and constitutes the dominant client-side compression cost. After selecting rank $\ell^{*}$, quantizing the retained singular vectors and singular values requires $O\;\left(\ell^{*}\left(m_{p}+n_{p}\right)\right)$ operations. Reconstructing the dense update at the server requires $O\;\left(m_{p}n_{p}\ell^{*}\right)$ operations. For a one-dimensional parameter with $h_{p}$ elements, direct quantization and reconstruction both require $O(h_{p})$ operations. Therefore, the additional computation depends primarily on the dimensions of the matrix-shaped parameters and their selected ranks, whereas the quantization cost is linear in the number of transmitted values.

### 5.4. Classification-Head Protection

The classifier head directly controls the decision boundaries between historical and newly introduced attack categories and is therefore especially sensitive to compression distortion. For each classifier-head tensor *p*, EdgeFedCIL evaluates the relative reconstruction error

(25) $$\varepsilon_{k,t,r}^{p}=\frac{{∥u_{k,t,r}^{p}-{\hat{u}}_{k,t,r}^{p}∥}_{2}^{2}}{{∥u_{k,t,r}^{p}∥}_{2}^{2}+\epsilon }.$$

If this error exceeds the admissible threshold $\eta_{\mathrm{head}}$, the compressed representation of that tensor is rejected and the compensated update is transmitted in full precision. Because the transmitted and target tensors are then identical, the corresponding residual is cleared. This selective fallback protects sensitive output parameters while allowing the feature-extraction layers to remain compressed.

### 5.5. Server Reconstruction and Weighted Aggregation

The server reconstructs every received parameter representation and assembles the reconstructed error-feedback-compensated update ${\hat{u}}_{k,t,r}$. The aggregation weight of client *k* is proportional to $n_{k,t}^{\mathrm{real}}$, the number of distinct current real samples and retained real exemplars used before synthetic balancing. In other words, $a_{k,t,r}=n_{k,t}^{\mathrm{real}}/\sum_{j}n_{j,t}^{\mathrm{real}}$ over clients that successfully returned an update. Synthetic samples therefore cannot increase a client’s influence on the global model.

The server update is

(26) $$\theta_{t,r+1}=\theta_{t,r}+\sum_{k\in S_{t,r}^{+}}a_{k,t,r}{\hat{u}}_{k,t,r},$$

where the summation covers the selected clients that completed local training and returned valid payloads. Their weights are normalized to sum to one. The reconstruction and aggregation process changes neither the ownership of local data nor the exemplar memories maintained in Section 4.

### 5.6. Compression and Aggregation Procedure

Algorithm 2 summarizes the communication module invoked once per federated round by Algorithm 1. Compression is enabled from phase 1 onward. Residuals are client-specific and parameter-specific, so approximation errors from different clients or layers are never mixed.
**Algorithm 2** EdgeFedCIL Update Compression and Server Aggregation**Require:**
Global model $\theta_{t,r}$, local models $\left\{\theta_{t,r}^{k}\right\}$, real-sample counts, residual buffers **Ensure:**
Updated global model $\theta_{t,r+1}$ and residual buffers
1:**for all** $k\in S_{t,r}^{+}$ in parallel **do**2:      $\Delta \theta_{k,t,r}\leftarrow \theta_{t,r}^{k}-\theta_{t,r}$3:      **for all** parameter tensors *p* **do**4:            $u_{k,t,r}^{p}\leftarrow \Delta \theta_{k,t,r}^{p}+e_{k,t,r-1}^{p}$5:            **if** $u_{k,t,r}^{p}$ is two-dimensional **then**6:                Select $\ell^{*}$ using (18) and quantize the low-rank factors7:            **else**8:                  Quantize $u_{k,t,r}^{p}$ directly9:            **end if**10:            Reconstruct ${\hat{u}}_{k,t,r}^{p}$ from the encoded representation11:            **if** *p* belongs to the classifier head and $\varepsilon_{k,t,r}^{p}>\eta_{\mathrm{head}}$ **then**12:                  Transmit $u_{k,t,r}^{p}$ in full precision and set ${\hat{u}}_{k,t,r}^{p}\leftarrow u_{k,t,r}^{p}$13:            **end if**14:            Update $e_{k,t,r}^{p}$ using (17)15:      **end for**16:      Upload the encoded update and $n_{k,t}^{\mathrm{real}}$17:**end for**18:Reconstruct the valid client updates and update the global model using (26)19:**return** $\theta_{t,r+1}$ and the residual buffers


### 5.7. Phase-Wise Convergence Analysis

Because the observed classes and local objectives change between incremental phases, convergence is analyzed within a fixed phase *t*. Define the expected phase-level objective as

(27) $$F_{t}\left(\theta \right)=\sum_{k=1}^{K}\pi_{k,t}F_{k,t}\left(\theta \right),\;\sum_{k=1}^{K}\pi_{k,t}=1,$$

where $F_{k,t}$ is the expected local objective associated with (13). One communication round is represented as

(28) $$\theta_{t,r+1}=\theta_{t,r}-\eta_{t}\left(g_{t,r}+\xi_{t,r}\right),$$

where $g_{t,r}$ is the effective uncompressed federated descent direction and $\xi_{t,r}$ is the perturbation introduced by compression and reconstruction.

**Assumption 1.**  *For a fixed phase t, $F_{t}$ is lower bounded by $F_{t}^{★}$ and has an $L_{t}$-Lipschitz continuous gradient. The effective federated direction and compression perturbation satisfy*

(29) $$E\;\left[{∥g_{t,r}-\nabla F_{t}\left(\theta_{t,r}\right)∥}_{2}^{2}\;|\;\theta_{t,r}\right]\leq \sigma_{t}^{2},\;E\;\left[{∥\xi_{t,r}∥}_{2}^{2}\;|\;\theta_{t,r}\right]\leq \delta_{t}^{2}.$$

Here, $\sigma_{t}^{2}$ captures stochastic local optimization, partial participation, multiple local updates, and client heterogeneity. The compression term $\delta_{t}^{2}$ is bounded by the reconstruction properties established in Section 5. In particular, the low-rank truncation bound in (19), bounded quantization error, and classifier-head fallback jointly provide a finite relative reconstruction-error bound. If the compensated client updates have bounded second moment $G_{t}^{2}$, then Jensen’s inequality gives $\delta_{t}^{2}\leq \beta_{t}G_{t}^{2}$ for a finite compression factor $\beta_{t}$. Moreover, the residual update in (17) carries the current reconstruction error into later communication rounds.

**Proposition 2**  (Phase-wise stationarity). *Under the stated assumption, if $0<\eta_{t}\leq 1/\left(6L_{t}\right)$, then after $R_{t}$ communication rounds,*

(30) $$\frac{1}{R_{t}}\sum_{r=0}^{R_{t}-1}E\left[{∥\nabla F_{t}\left(\theta_{t,r}\right)∥}_{2}^{2}\right]\leq \frac{4\left(F_{t}\left(\theta_{t,0}\right)-F_{t}^{★}\right)}{\eta_{t}R_{t}}+4\left(1+\frac{3L_{t}\eta_{t}}{2}\right)\left(\sigma_{t}^{2}+\delta_{t}^{2}\right).$$

**Proof.**  Let $d_{t,r}=g_{t,r}-\nabla F_{t}\left(\theta_{t,r}\right)$. Applying $L_{t}$-smoothness to (28), together with $\left|\langle a,b\rangle \right|\leq {∥a∥}_{2}^{2}/4+{∥b∥}_{2}^{2}$ and ${∥a+b+c∥}_{2}^{2}\leq {3∥a∥}_{2}^{2}+{3∥b∥}_{2}^{2}+3{∥c∥}_{2}^{2}$, gives

(31) $$\begin{array}{cc} E\left[F_{t}\left(\theta_{t,r+1}\right)\;|\;\theta_{t,r}\right]\leq \;\; & F_{t}\left(\theta_{t,r}\right)-\left(\frac{\eta_{t}}{2}-\frac{3L_{t}\eta_{t}^{2}}{2}\right){∥\nabla F_{t}\left(\theta_{t,r}\right)∥}_{2}^{2}\\ \; & +\left(\eta_{t}+\frac{3L_{t}\eta_{t}^{2}}{2}\right)\left(\sigma_{t}^{2}+\delta_{t}^{2}\right). \end{array}$$

Since $\eta_{t}\leq 1/\left(6L_{t}\right)$, the coefficient of the gradient term is at least $\eta_{t}/4$. Summing over $r=0,\ldots ,R_{t}-1$, using the lower bound $F_{t}^{★}$, and dividing by $\eta_{t}R_{t}/4$ yields (30). □

The first term in (30) decreases with the number of communication rounds, whereas $\sigma_{t}^{2}$ and $\delta_{t}^{2}$ determine the stationary neighborhood caused by federated optimization and compression. Since the objective changes when new attack classes are introduced, the result applies separately to each incremental phase rather than to a single stationary solution over the complete class-incremental sequence.

## 6. Experimental Evaluation

This section evaluates EdgeFedCIL on two IoT intrusion-detection datasets. The analysis focuses on final detection performance, historical-knowledge retention, convergence behavior, component effectiveness, and client-to-server communication cost.

### 6.1. Experimental Setup

#### 6.1.1. Datasets and Preprocessing

The ToN-IoT [40] and X-IIoTID [41] datasets are used to evaluate the method under different traffic distributions. The four incremental phases are denoted by P1–P4. Six classes are retained from each dataset and organized into four incremental phases, as shown in Table 2. For both datasets, identifier, timestamp, constant, duplicate, and label-leakage attributes are removed. Numerical features are standardized, and categorical features in X-IIoTID are encoded before training. Each dataset is divided class-wise into training, validation, and test subsets with an approximate ratio of 80%–10%–10%. All methods use identical preprocessing outputs and data splits.

#### 6.1.2. Comparison Methods and Basic Settings

EdgeFedCIL is compared with FedAvg [5], FedProx [42], Fed-LwF [30], Fed-EWC [43], and GLFC [33]. FedAvg provides the standard aggregation baseline, FedProx constrains local model drift, Fed-LwF preserves previous outputs through KD, and Fed-EWC regularizes parameters that are important to earlier phases. As a recent FCIL baseline, GLFC mitigates local and global forgetting through class-aware gradient compensation, class-semantic relation distillation, and global-model selection. All methods use the same Transformer-style classifier, data stream, client partition, client-selection sequence, optimizer, and validation-based checkpoint-selection protocol.

To evaluate communication efficiency under compressed transmission, EdgeFedCIL is further compared with PowerSGD [27], Top-K sparsification [28], and SignSGD [44]. These methods represent three commonly used compression strategies: low-rank approximation, sparse update transmission, and sign-based low-bit encoding, respectively. Each communication baseline is applied to the same client model differences and uses the same continual-learning procedure, model architecture, client partitions, and training configuration as EdgeFedCIL. Thus, the comparison isolates the effect of the communication-compression strategy. The compression parameters of PowerSGD and Top-K are selected to produce cumulative upload volumes comparable to that of EdgeFedCIL, while the lower communication cost of SignSGD is reported using one sign bit per coordinate together with a per-tensor mean-absolute-value scale.

The classifier receives each preprocessed feature vector after zero padding to 1024 dimensions and reshapes it into eight tokens with an embedding dimension of 128. The encoder contains three Transformer-style blocks. Each block uses eight-head self-attention, with a dimension of 16 for each attention head, followed by a position-wise feed-forward network with a hidden dimension of 512. Residual connections and layer normalization are applied after both the attention and feed-forward operations, and the attention dropout rate is set to 0.1. The output tokens are flattened into a 1024-dimensional representation and passed to a linear classifier with six output units. The complete model contains 551,430 trainable parameters.

Following the protocol defined in Section 3, the experiments use 20 clients, a participation ratio of 0.6, 50 communication rounds per phase, and two local epochs per selected client. The Dirichlet concentration is varied over α∈{0.1,0.5,1.0}. Adam is used with a learning rate of $10^{-4}$ and a mini-batch size of 128. The local balancing parameters are $\eta_{\mathrm{bal}}=0.5$ and κ=5. For EdgeFedCIL, the exemplar ratio is 0.1 with a maximum of 500 exemplars per client, the new-class weight is 2, and the KD temperature and weight are 2 and 0.1. Communication compression retains 97% spectral energy and uses 8-bit quantization. The base random seed is set to 42. The parameter-regularization coefficient is $\lambda_{2}=0.0015$. When the memory budget permits, at least 50 exemplars are retained for each locally represented class. The reconstruction-error threshold for classifier-head protection is set to 0.2. Upload volume is reported in megabytes (MB) and includes client-to-server model information but excludes protocol headers and server broadcasts.

All experiments were implemented in Python 3.10.19 using PyTorch 2.10.0+cu128 and CUDA 12.8, and were conducted on a workstation equipped with an AMD Ryzen 9 9955HX 16-Core Processor CPU, 16 GB of RAM, and an NVIDIA GeForce RTX 5070 Laptop GPU with 8 GB of GPU memory. Client-side encoding time was measured under the same hardware and software environment and averaged over all participating client uploads.

#### 6.1.3. Evaluation Metrics

The reported detection metrics are accuracy, macro-averaged F1 score (Macro-F1), old-class Macro-F1, and, in the phase-wise analysis, new-class Macro-F1. Historical retention is measured by forgetting and backward transfer (BWT), while communication efficiency is measured by cumulative client-to-server upload.

Let $t_{c}$ denote the phase in which class *c* is first introduced, and let $q_{t,c}$ denote its recall on the held-out test set after phase *t*. For t>1, average forgetting is defined as

(32) $${\mathrm{Forgetting}}_{t}=\frac{1}{|C_{t}^{\mathrm{old}}|}\sum_{c\in C_{t}^{\mathrm{old}}}{\left[\max_{\tau \in \{t_{c},\ldots ,t-1\}}q_{\tau ,c}-q_{t,c}\right]}_{+}.$$

Let $A_{t,j}$ denote the Accuracy on the classes introduced in phase *j*, evaluated after phase *t*. For t>1, BWT is

(33) $${\mathrm{BWT}}_{t}=\frac{1}{t-1}\sum_{j=1}^{t-1}\left(A_{t,j}-A_{j,j}\right).$$

Lower forgetting and BWT closer to zero indicate better retention.

### 6.2. Main Experimental Results

Table 3 and Table 4 report the final-phase results under all non-IID settings. The reported upload is accumulated over the four incremental phases.

On ToN-IoT, EdgeFedCIL achieves the best Accuracy, Macro-F1, old-class Macro-F1, and forgetting under all three values of α. Under the strongest heterogeneity setting (α=0.1), it outperforms GLFC by 6.72 percentage points in Accuracy and 9.01 points in Macro-F1, while reducing forgetting from 0.1187 to 0.0616. On X-IIoTID, EdgeFedCIL also achieves the best results at α=0.1, exceeding GLFC by 0.87 points in Accuracy and 0.49 points in Macro-F1. Under the milder α=0.5 and 1.0 settings, GLFC obtains marginally higher detection performance and lower forgetting, whereas EdgeFedCIL maintains substantially lower cumulative upload. These results indicate that EdgeFedCIL provides a favorable balance between detection performance, historical-knowledge retention, and communication cost, particularly under highly heterogeneous client distributions.

### 6.3. Convergence and Class-Level Analysis

**Figure 2:**
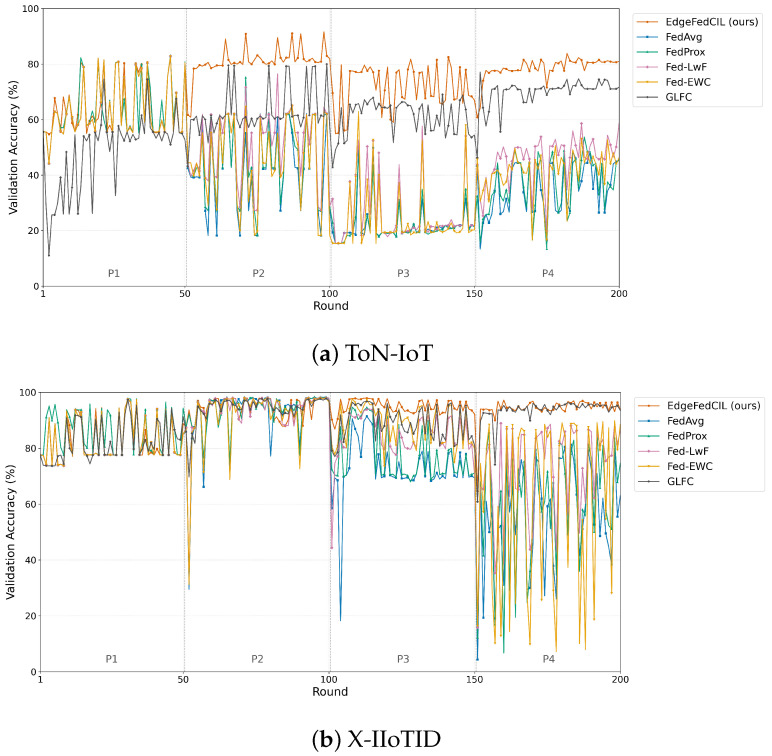
Validation Accuracy over 4 phases (200 communication rounds) under α=0.1. Dashed lines indicate phase boundaries.

Figure 2 shows that EdgeFedCIL recovers rapidly after phase transitions and remains more stable during the later phases. The separation is especially clear on ToN-IoT after P2, where the baselines fluctuate at substantially lower levels. On X-IIoTID, most methods perform well in the early phases, but EdgeFedCIL maintains a narrow high-Accuracy range after the additional classes are introduced.

The phase-wise results in Table 5 show that the main ToN-IoT difficulty occurs in P3, where the new-class F1 falls to 68.07% after the password class is introduced. Performance partially recovers in P4, and the ransomware class reaches 100% F1. X-IIoTID remains stable across all phases, with Macro-F1 above 96% and final forgetting of only 0.0069.

**Figure 3:**
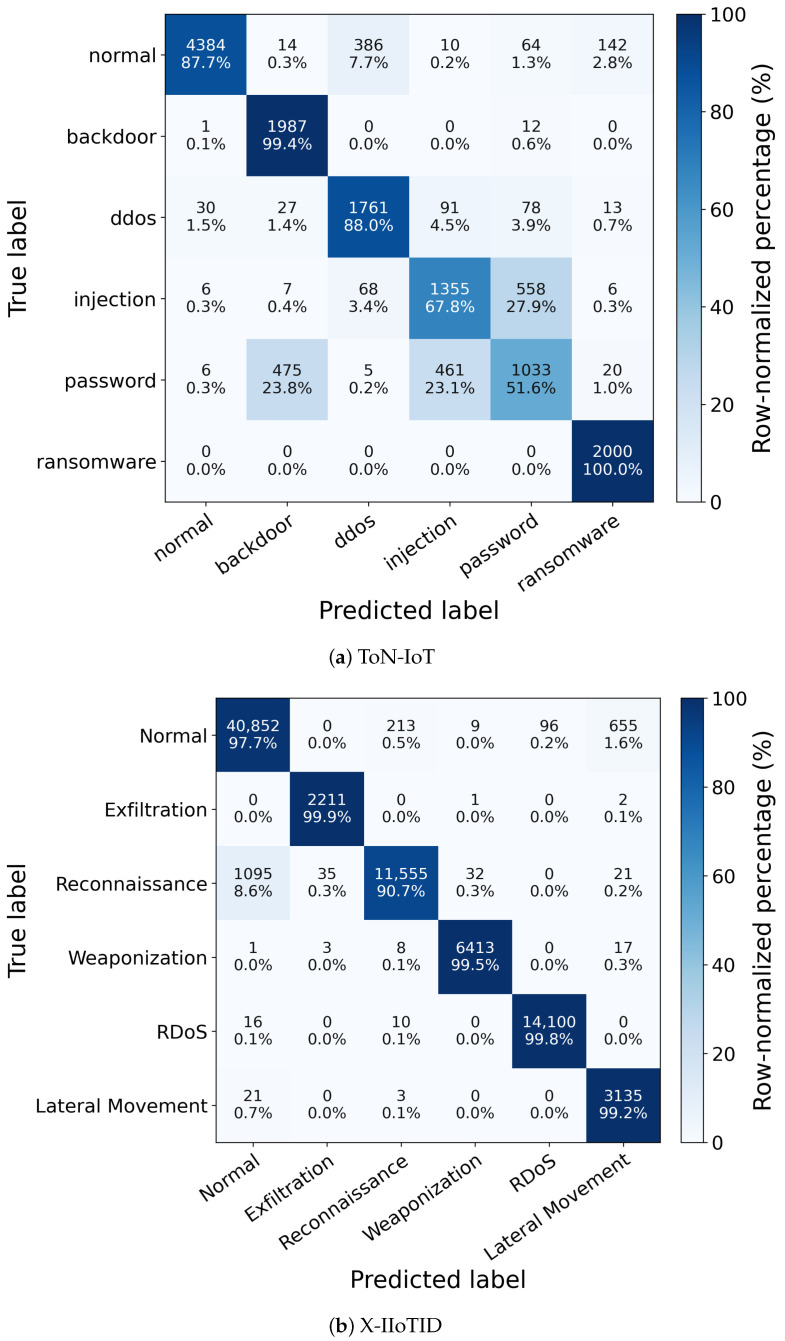
Row-normalized final-phase confusion matrices of EdgeFedCIL under α=0.1.

Figure 3 indicates that the remaining ToN-IoT errors are concentrated between injection and password traffic, whereas backdoor and ransomware are recognized almost perfectly. On X-IIoTID, every class recall exceeds 90%; the main residual error is Reconnaissance traffic being predicted as Normal. The concentrated error patterns are consistent with the phase-wise Macro-F1 results.

### 6.4. Historical-Knowledge Retention

**Figure 4:**
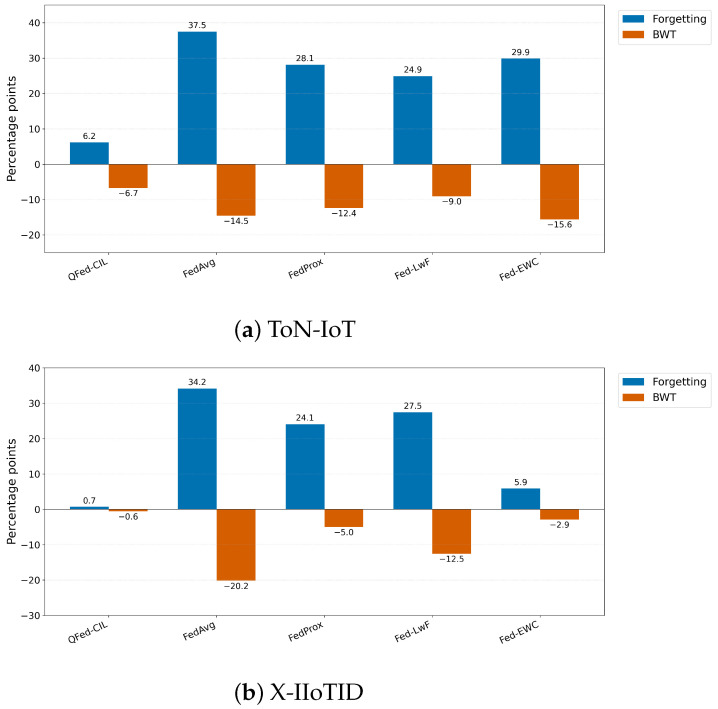
Final-phase forgetting and BWT under α=0.1. Values are shown in percentage points.

Figure 4 shows that EdgeFedCIL achieves the lowest forgetting and the BWT closest to zero among the methods shown. Its forgetting is 6.2 percentage points on ToN-IoT and only 0.7 percentage points on X-IIoTID. The corresponding BWT values of −6.7 and −0.6 percentage points confirm that EdgeFedCIL limits historical-task degradation while learning new classes.

**Figure 5:**
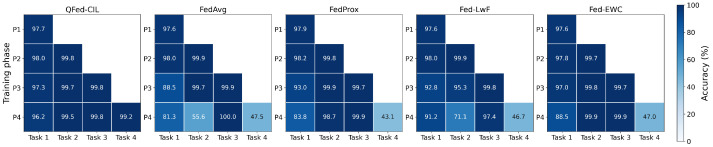
Task-Accuracy matrices on X-IIoTID under α=0.1. Entry (t,j) is the Accuracy on task *j* after phase *t*.

The task matrices in Figure 5 further show that EdgeFedCIL preserves the first three tasks at 96.2%, 99.5%, and 99.8% after P4 while attaining 99.2% on the newest task. The baselines either lose more accuracy on earlier tasks or fail to learn the final task sufficiently, illustrating the stability–plasticity balance provided by replay and KD.

### 6.5. Ablation Study

Table 6 reports the final-phase ablation results on ToN-IoT under α=0.1, where the class-incremental problem is most challenging. The full model is compared with variants without communication compression, exemplar replay, or KD.

Removing replay causes the largest degradation, reducing Macro-F1 by 32.83 percentage points and increasing forgetting from 0.0616 to 0.2709. Removing KD also weakens historical-class performance, confirming its complementary stabilizing role. Disabling compression increases cumulative upload from 634.64 MB to 5048.49 MB without improving detection performance, showing that the compression module provides substantial communication savings without sacrificing the effectiveness of the continual-learning framework.

### 6.6. Sensitivity Analysis

We conduct a sensitivity analysis on ToN-IoT under α=0.1 to examine the effects of the spectral-energy threshold τ, the KD weight $\lambda_{\mathrm{kd}}$, and the new-class weight $\omega_{\mathrm{new}}$. Each experiment varies one parameter while keeping the remaining settings unchanged. Table 7 reports the final detection performance, historical-class retention, forgetting, and cumulative client-to-server upload.

The spectral-energy threshold produces the clearest communication–performance trade-off. Reducing τ from 0.97 to 0.90 decreases cumulative upload from 634.64 MB to 484.04 MB, but also reduces Accuracy and Macro-F1 and increases forgetting. Increasing τ to 0.99 substantially increases the transmitted volume without improving detection or historical-class retention. Thus, τ=0.97 provides the most favorable balance among detection performance, forgetting, and communication cost.

The two KD weights produce similar final detection performance. Although $\lambda_{\mathrm{kd}}=0.20$ yields a marginally higher Macro-F1, $\lambda_{\mathrm{kd}}=0.10$ achieves lower forgetting and cumulative upload. For the new-class weight, $\omega_{\mathrm{new}}=2$ provides the best overall balance. A weight of 1 produces slightly lower detection performance, whereas increasing the weight to 3 excessively emphasizes new-class adaptation and leads to a clear degradation in historical-class performance. Overall, the default settings remain stable across moderate parameter variations and provide a balanced configuration for detection, knowledge retention, and communication efficiency.

### 6.7. Communication-Efficiency Analysis

Across all evaluated settings, EdgeFedCIL requires substantially less cumulative client-to-server upload than transmitting the same client model differences in full precision. Under α=0.1, the cumulative upload decreases from 5048.49 MB to 634.64 MB on ToN-IoT and from 4989.59 MB to 473.51 MB on X-IIoTID, corresponding to reductions by factors of approximately 7.95 and 10.54, respectively. These results demonstrate that the proposed communication mechanism substantially reduces the repeated client-to-server transmission incurred across incremental phases.

To evaluate the effectiveness of the adaptive communication strategy, EdgeFedCIL is compared with PowerSGD, Top-K, and SignSGD on ToN-IoT under α=0.1. The methods share the same continual-learning procedure, model architecture, client partitions, client-participation sequence, and optimization settings, and differ in the client-update encoding strategy and the corresponding server-side reconstruction or aggregation procedure. Table 8 reports the final detection performance, historical-class retention, transmitted data volume, and client-side encoding time.

For the communication baselines, PowerSGD uses a fixed rank of r=10 for matrix updates, while Top-K retains the largest 5% of update coordinates. SignSGD transmits one sign bit per coordinate and applies sample-size-weighted FedAvg aggregation at the server after reconstructing the signed updates with a per-tensor mean-absolute-value scale.

PowerSGD and Top-K achieve per-client communication volumes comparable to that of EdgeFedCIL, while SignSGD provides a lower per-client upload through one-bit update representation. Under these compressed-transmission settings, EdgeFedCIL achieves the highest Accuracy, Macro-F1, and old-class Macro-F1, as well as the lowest forgetting among the compared methods. EdgeFedCIL requires 134.03 ms of encoding time per participating client, which is close to the 129.86 ms required by PowerSGD, while providing higher detection performance and lower forgetting. In particular, compared with PowerSGD, which has a closely matched upload per participating client, EdgeFedCIL improves Accuracy and Macro-F1 by 3.80 and 3.67 percentage points, respectively, while reducing forgetting from 0.1286 to 0.0616. This advantage is consistent with the coordinated operation of adaptive low-rank approximation, low-bit quantization, error feedback, and classifier-head protection. Adaptive rank selection preserves the dominant spectral information of each update, quantization reduces the representation cost of the retained factors, error feedback reintroduces discarded information in subsequent rounds, and classifier-head protection limits excessive distortion of class-discriminative parameters. These mechanisms jointly provide a more favorable detection–communication trade-off than the representative low-rank, sparsification-based, and sign-based compression baselines.

The average upload of EdgeFedCIL is approximately 0.2644 MB per participating client in each communication round, indicating a low communication burden. This reduced transmission volume supports the applicability of the proposed framework to bandwidth-constrained edge IoT environments from a communication perspective.

## 7. Discussion

The experiments use ToN-IoT and X-IIoTID, which provide different traffic distributions, attack categories, and IoT application settings. Evaluations under multiple non-IID levels further examine the framework under different degrees of client heterogeneity. Nevertheless, two public datasets cannot fully represent the diversity of operational edge IoT networks, where device behavior, feature availability, attack prevalence, and label quality may vary. Therefore, the reported results provide controlled benchmark evidence, while evaluation on additional datasets and continuously collected traffic from real deployments remains necessary.

The scalability of EdgeFedCIL depends on the numbers of participating clients, communication rounds, attack classes, and incremental phases. For a fixed model architecture, the communication volume of an individual participating client is mainly determined by the adaptively selected ranks and does not directly increase with the total number of clients. However, the aggregate communication grows with the number of participating clients. Increasing the communication rounds or incremental phases also increases cumulative upload because compressed updates are repeatedly transmitted. As the number of attack classes increases, the classifier head expands, while a fixed exemplar-memory budget provides fewer retained samples for each historical class. These factors may increase both communication demand and the difficulty of historical-knowledge retention.

From a communication perspective, EdgeFedCIL requires an average upload of approximately 0.2644 MB per participating client in each communication round under the evaluated setting. Compared with representative low-rank, sparsification-based, and sign-based compression methods, EdgeFedCIL achieves higher detection performance and lower forgetting under comparable compressed-transmission settings. These results support its applicability to bandwidth-constrained edge IoT environments. Although the current implementation employs exact singular value decomposition to ensure accurate spectral-energy estimation and adaptive rank selection, approximate or randomized SVD techniques could further reduce client-side compression latency and computational overhead on highly resource-constrained edge devices, particularly when larger models are deployed. However, the resulting approximation error may affect rank selection, update reconstruction, and continual-learning performance. Future work will therefore investigate the trade-offs among approximation accuracy, computational cost, communication efficiency, and historical-knowledge retention, while further evaluating the framework on additional datasets and real edge devices with larger client populations and longer incremental attack streams.

## 8. Conclusions

This paper proposed EdgeFedCIL, a communication-efficient federated class-incremental intrusion detection framework for dynamic and heterogeneous edge IoT environments. EdgeFedCIL jointly addresses catastrophic forgetting, new-class plasticity, and cumulative communication overhead during the continual acquisition of emerging attack knowledge. At the learning level, client-local exemplar replay provides direct supervision for previously observed classes, knowledge distillation stabilizes historical output relationships, and new-class-weighted classification strengthens adaptation to newly introduced attacks. At the communication level, adaptive low-rank matrix approximation and low-bit quantization reduce the size of uploaded model differences, while error feedback and classifier-head protection limit the loss of class-discriminative information caused by compression. Experiments on ToN-IoT and X-IIoTID under multiple non-IID settings showed several consistent trends. EdgeFedCIL achieved competitive or superior detection and historical-knowledge retention performance across the evaluated settings while substantially reducing client-to-server communication. Its advantage was particularly evident under highly heterogeneous client distributions, where conventional federated and continual-learning methods were more vulnerable to local class imbalance, model drift, and historical-knowledge degradation. The ablation results further showed that exemplar replay and knowledge distillation play complementary roles in historical-knowledge preservation, while new-class weighting supports model plasticity. Meanwhile, the matrix-compression mechanism substantially reduced cumulative client-to-server communication without degrading the effectiveness of the continual-learning process. Overall, EdgeFedCIL provides a practical balance among historical-class stability, new-class plasticity, and communication efficiency, supporting reliable continual intrusion detection as edge IoT traffic distributions and attack classes evolve over time.

## Figures and Tables

**Table 1:** Principal Notation Used in This Paper.

Symbol	Description	Symbol	Description
K,k	Total number of clients and client index	T,t,r	Total number of phases, phase index, and communication-round index
$\theta_{t,r}$	Global model parameters at phase *t* and round *r*	$\theta_{t,r}^{k}$	Locally trained model parameters of client *k*
$\Delta \theta_{k,t,r}$	Local model difference produced by client *k*	C	Complete selected label space
$C_{t}^{\mathrm{new}}$	Classes introduced for the first time in phase *t*	$C_{t}^{\mathrm{seen}}$	Classes observed by the end of phase *t*
$C_{t}^{\mathrm{old}}$	Classes introduced before phase *t*	$D_{k,t}$	Real stream samples assigned to client *k* in phase *t*
$A_{k,t}$	Client-local sample set before class balancing	$B_{k,t}$	Client-local optimization multiset after class balancing
α,ρ	Dirichlet concentration and client participation ratio	$\eta_{\mathrm{bal}},\;\kappa$	Local balancing ratio and maximum expansion factor
$M_{k,t},\;M_{k,t}$	Client memory and its exemplar budget	$\omega_{\mathrm{new}}$	Classification weight assigned to new-class samples
$T_{\mathrm{kd}},\;\lambda_{\mathrm{kd}}$	KD temperature and KD-loss coefficient	$\lambda_{2}$	Parameter-regularization coefficient
$u_{k,t,r}^{p}$	Error-feedback-compensated update of parameter tensor *p*	$e_{k,t,r}^{p}$	Compression residual of parameter tensor *p*
$\tau ,\;\ell^{*}$	Spectral-energy threshold and selected low rank	$Q_{b}\left(\cdot \right)$	Symmetric *b*-bit quantization operator
$\eta_{\mathrm{head}}$	Classifier-head full-precision fallback threshold		

**Table 2:** Newly Introduced Classes in Each Incremental Phase.

Phase	ToN-IoT	X-IIoTID
P1	normal, backdoor, DDoS	Normal, Exfiltration, Reconnaissance
P2	injection	Weaponization
P3	password	RDoS
P4	ransomware	Lateral Movement

**Table 3:** Final-Phase Results on ToN-IoT.

α	Method	Accuracy (%)	Macro-F1 (%)	Old Macro-F1 (%)	Forgetting	Upload (MB)
0.1	FedAvg	48.61	30.10	26.41	0.3748	4884.42
	FedProx	53.39	40.13	38.32	0.2811	4884.42
	Fed-LwF	58.49	45.94	44.94	0.2492	4884.42
	Fed-EWC	52.53	41.25	39.61	0.2987	4884.42
	GLFC	76.75	71.76	69.07	0.1187	5051.51
	**EdgeFedCIL (ours)**	**83.47**	**80.77**	**77.79**	**0.0616**	**634.64**
0.5	FedAvg	55.87	43.71	42.26	0.4604	5048.49
	FedProx	56.21	44.71	43.46	0.4527	5048.49
	Fed-LwF	57.34	43.25	54.01	0.2235	5048.49
	Fed-EWC	54.48	42.97	41.68	0.4703	5048.49
	GLFC	75.06	73.64	73.35	0.1709	5051.51
	**EdgeFedCIL (ours)**	**77.51**	**76.76**	**77.11**	**0.1067**	**888.90**
1.0	FedAvg	63.04	55.65	52.04	0.3997	5048.49
	FedProx	58.30	46.47	40.77	0.4958	5048.49
	Fed-LwF	63.65	53.82	56.04	0.3356	5048.49
	Fed-EWC	58.83	52.22	52.78	0.4074	5048.49
	GLFC	78.48	74.21	71.71	0.1638	5051.51
	**EdgeFedCIL (ours)**	**80.57**	**78.64**	**77.42**	**0.1318**	**900.68**

**Table 4:** Final-Phase Results on X-IIoTID.

α	Method	Accuracy (%)	Macro-F1 (%)	Old Macro-F1 (%)	Forgetting	Upload (MB)
0.1	FedAvg	81.18	59.52	64.48	0.3415	4989.59
	FedProx	86.24	66.81	69.66	0.2407	4989.59
	Fed-LwF	88.96	66.21	73.16	0.2746	4989.59
	Fed-EWC	89.78	83.40	90.98	0.0589	4989.59
	GLFC	96.35	96.10	96.91	0.0104	4992.61
	**EdgeFedCIL (ours)**	**97.22**	**96.59**	**97.97**	**0.0069**	**473.51**
0.5	FedAvg	96.74	95.60	97.76	0.0181	5048.49
	FedProx	96.85	95.80	97.88	0.0169	5048.49
	Fed-LwF	97.41	96.86	98.13	0.0116	5048.49
	Fed-EWC	97.01	96.06	97.80	0.0151	5048.49
	GLFC	**97.83**	**97.43**	**98.44**	**0.0042**	5051.51
	**EdgeFedCIL (ours)**	97.51	96.96	98.21	0.0094	**539.96**
1.0	FedAvg	96.29	94.88	97.23	0.0227	5048.49
	FedProx	96.50	95.27	97.32	0.0210	5048.49
	Fed-LwF	96.40	95.02	97.24	0.0211	5048.49
	Fed-EWC	96.60	95.17	97.62	0.0167	5048.49
	GLFC	**97.76**	**97.34**	**98.36**	**0.0076**	5051.51
	**EdgeFedCIL (ours)**	97.45	96.79	98.14	0.0107	**548.86**

**Table 5:** Phase-Wise EdgeFedCIL Results Under α=0.1.

Dataset	Phase	Accuracy (%)	Macro-F1 (%)	Old Macro-F1 (%)	New Macro-F1 (%)	Forgetting
ToN-IoT	P1	83.54	78.67	–	78.67	0.0000
	P2	91.23	90.72	90.68	97.33	0.0000
	P3	82.06	78.24	88.82	68.07	0.0645
	P4	83.47	80.77	77.79	100.00	0.0616
X-IIoTID	P1	97.70	97.66	–	97.66	0.0000
	P2	98.18	98.27	97.81	99.88	0.0000
	P3	97.97	98.08	97.69	99.91	0.0045
	P4	97.22	96.59	97.97	99.62	0.0069

**Table 6:** Final-Phase Ablation Results on ToN-IoT Under α=0.1.

Method	Accuracy (%)	Macro-F1 (%)	Old Macro-F1 (%)	Forgetting	Cumulative Upload (MB)
Without Communication Compression	81.63	76.84	73.07	0.0863	5048.49
Without Replay	59.20	47.94	47.34	0.2709	580.14
Without KD	77.93	70.37	67.07	0.1601	627.06
**EdgeFedCIL (ours)**	**83.47**	**80.77**	**77.79**	**0.0616**	**634.64**

**Table 7:** Sensitivity analysis of key hyperparameters on ToN-IoT under α=0.1. The superscript * denotes the default setting.

Parameter	Value	Accuracy (%)	Macro-F1 (%)	Old Macro-F1 (%)	Forgetting	Cumulative Upload (MB)
Spectral-energy threshold τ	0.90	79.55	77.04	77.23	0.0831	484.04
	0.97 ^∗^	83.47	80.77	77.79	0.0616	634.64
	0.99	80.84	76.15	72.25	0.0951	841.89
KD weight $\lambda_{\mathrm{kd}}$	0.10 ^∗^	83.47	80.77	77.79	0.0616	634.64
	0.20	83.41	80.79	77.80	0.0687	653.46
New-class weight $\omega_{\mathrm{new}}$	1	83.21	80.39	77.32	0.0642	644.77
	2 ^∗^	83.47	80.77	77.79	0.0616	634.64
	3	81.19	74.29	70.01	0.0946	648.17

**Table 8:** Comparison with Representative Communication-Compression Baselines on ToN-IoT Under α=0.1

Method	Accuracy (%)	Macro-F1 (%)	Old Macro-F1 (%)	Forgetting	Upload (MB/Client/Round)	Encoding Time (ms/Client)
PowerSGD	79.67	77.10	75.12	0.1286	0.2698	129.86
Top-K	81.19	74.30	70.02	0.1111	0.2320	62.49
SignSGD	79.73	77.32	77.57	0.0748	0.0826	91.41
EdgeFedCIL (ours)	83.47	80.77	77.79	0.0616	0.2644	134.03

## Data Availability

The datasets used in this study are publicly available. The X-IIoTID dataset can be accessed from its official repository at https://github.com/Alhawawreh/X-IIoTID (accessed on 30 June 2026) and its Kaggle page at https://www.kaggle.com/datasets/munaalhawawreh/xiiotid-iiot-intrusion-dataset (accessed on 30 June 2026). The ToN-IoT dataset can be accessed from the official UNSW Research dataset page at https://research.unsw.edu.au/projects/toniot-datasets (accessed on 30 June 2026). The implementation code will be made available by the corresponding author upon reasonable request.
